# Inhibition of IRE1α RNase activity sensitizes patient‐derived acute myeloid leukaemia cells to proteasome inhibitors

**DOI:** 10.1111/jcmm.17479

**Published:** 2022-07-13

**Authors:** Stuart Creedican, Claire M. Robinson, Katarzyna Mnich, Md Nahidul Islam, Eva Szegezdi, Ruth Clifford, Janusz Krawczyk, John B. Patterson, Stephen P. FitzGerald, Mark Summers, Ciaran Richardson, Kenneth Martin, Adrienne M. Gorman, Afshin Samali

**Affiliations:** ^1^ Apoptosis Research Centre University of Galway Galway Ireland; ^2^ School of Biological and Chemical Sciences University of Galway Galway Ireland; ^3^ Randox Teoranta Dungloe, Co. Donegal Ireland; ^4^ Limerick Digital Cancer Research Centre, HRI, School of Medicine University of Limerick Limerick Ireland; ^5^ Department of Haematology University Hospital Limerick Limerick Ireland; ^6^ School of Medicine University of Galway Galway Ireland; ^7^ Department of Haematology Galway University Hospital Galway Ireland; ^8^ Orinove Inc. Newbury Park California USA; ^9^ Randox Laboratories Ltd Crumlin, Co. Antrim UK

To the Editor,

Despite improvements in prognostic stratification and optimization of therapeutic intervention in acute myeloid leukaemia (AML) patients, long‐term survival is low. Clinical trials suggest proteasome inhibitors may be beneficial, but further interrogation of the molecular consequences of proteasome inhibition in AML is warranted to identify novel approaches that enhance their efficacy.^1^ In multiple myeloma (MM), resistance to proteasome inhibitors can occur upon activation of the unfolded protein response (UPR), a stress response pathway that can control cell fate.^2^ Inositol‐requiring enzyme 1 alpha (IRE1α) is one of three stress sensors that mediates UPR signalling. IRE1α activity occurs via its RNase domain resulting in cleavage of a 26‐nucleotide intron from X‐Box Binding Protein 1 (XBP1) mRNA leading to formation of a transcription factor, XBP1s. XBP1s enhances cell survival by increasing transcription of genes associated with protein folding, endoplasmic reticulum‐associated degradation (ERAD) and phospholipid synthesis. We demonstrate that an IRE1 RNase inhibitor (MKC8866), in combination with proteasome inhibitors, significantly decreases XBP1s levels and increases cell death in AML cell lines and patient‐derived AML cells. In addition, this combination treatment can successfully target the CD34^+^CD38^−^ population and reduce clonogenic ability.

In some cancer types, targeting IRE1α alone may promote cell death.^3^ We investigated the impact of inhibiting basal IRE1 RNase activity with MKC8866 in three AML cell lines: U937, Molm 13 and KG1a. Expression of XBP1s protein was quantified using a previously described biochip array assay.^4^ In all cell lines, XBP1s was detected and was significantly decreased with MKC8866 treatment (Figure 1A, left panel). This had no impact on cell death (Figure 1A, right panel). Treatment with either bortezomib (BTZ) or carfilzomib (CFZ) increased XBP1s in KG1a and U937 cells. MKC8866 reversed this increase (Figure 1B,C, left panels). BTZ or CFZ caused cell death in KG1a and U937 cells and MKC8866 co‐treatment significantly enhanced AML cell death (Figure 1B,C, right panels).

**FIGURE 1:**
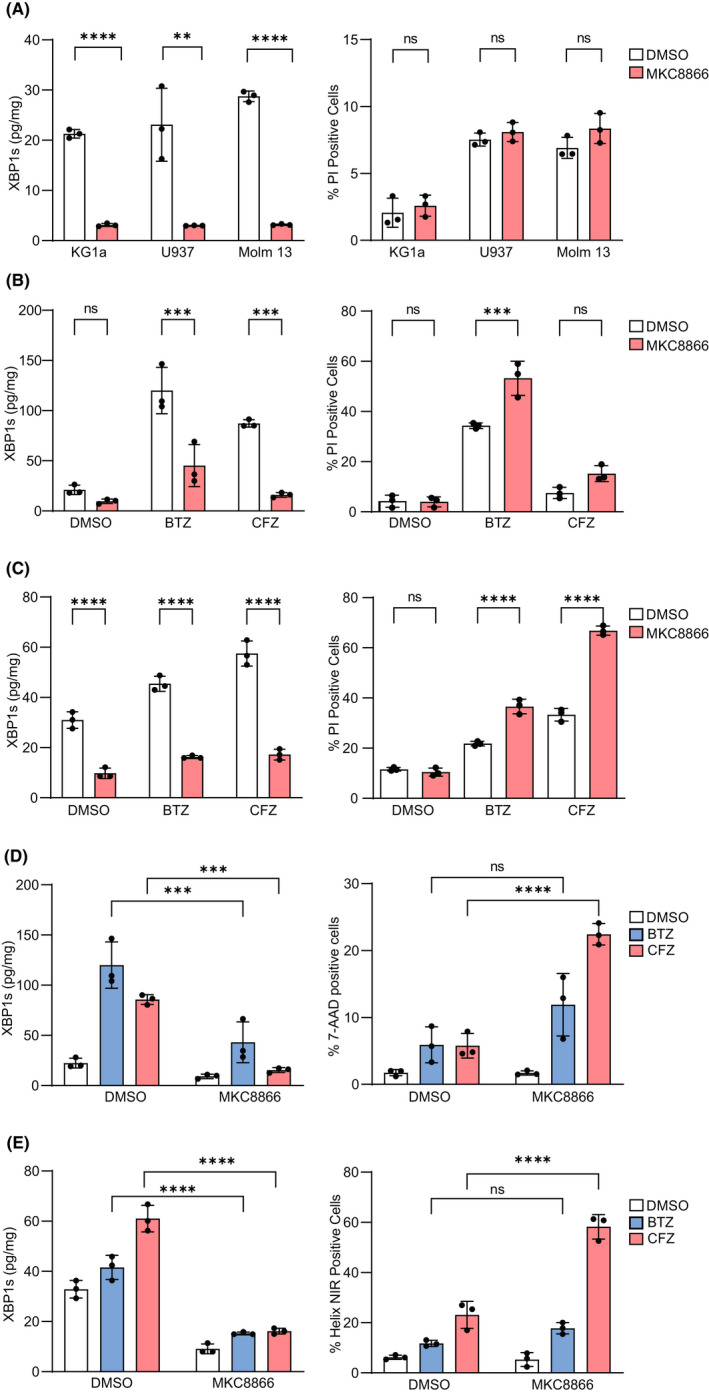
Targeting IRE1 RNase activity in AML cell lines in combination with proteasome inhibitors increases cell death. (A) Left panel: KG1a, U937 and Molm 13 cells were treated with dimethyl sulphoxide (DMSO) or 20 μM of the IRE1 RNase inhibitor MKC8866 for 16 h. XBP1s protein was assessed by XBP1 biochip.^4^ Right panel: KG1a, U937 and Molm 13 cells were treated with DMSO or 20 μM MKC8866 for 48 h. Cell death was assessed using propidium iodide (PI) and flow cytometry. (B, C) Left panels: KG1a (B) and U937 (C) cells were treated with DMSO or 10 nM BTZ or 20 nM CFZ ± 20 μM MKC8866 for 16 h and XBP1s was measured using XBP1s biochip. Right panels: KG1a (B) and U937 (C) cells were treated with DMSO, 10 nM BTZ or 20 nM CFZ ± 20 μM MKC8866 for 24 h (U937) or 48 h (KG1a) and assessed for PI uptake using flow cytometry. (D, E) Left panels: Co‐cultures of U937 + HS‐5 cells (D) and KG1a + HS‐5 (E) cells were treated for 24 and 48 h, respectively, with DMSO, 10 nM BTZ or 20 nM CFZ + 20 μM MKC8866 and XBP1s protein levels were assessed in AML cell lines using XBP1 biochip. Right panels: Co‐cultures of U937 + HS‐5 cells (D) and KG1a + HS‐5 cells were treated for 24 and 48 h, respectively, with DMSO, 10 nM BTZ or 20 nM CFZ + 20 μM MKC and were assessed for cell death quantified as a percentage of 7‐aminoactinomyc in D (7‐AAD) or helix NIR positive cells using flow cytometry. Experiments were performed *n* = 3, error bars = standard deviation. Unpaired *t*‐tests or one‐way anova with post‐hoc Tukey tests were used to determine statistical significance. ns = not significant, *p < 0.05, **p < 0.01, ***p < 0.001, ****p < 0.0001

Bone marrow mesenchymal stromal cells (BMSCs) protect AML cells from chemotherapy‐mediated killing, which can be modelled *in vitro* by co‐culture with HS‐5 cells, an immortalized BMSC feeder cell line.^5^ We hypothesized that BMSC would protect AML cells treated with proteasome inhibitor(s), and that IRE1α inhibitors may overcome this protection. KG1a and U937 cells were co‐cultured with HS‐5 cells and treated with BTZ or CFZ and DMSO or MKC8866. In both AML cell lines, MKC8866 reduced XBP1s levels in proteasome inhibitor‐treated cells (Figure 1D,E, left panels). Significantly increased AML cell death was observed in the combination treatments that included CFZ (*p* < 0.0001); increased cell death was observed in the combination treatments with BTZ but this was not significant (Figure 1D,E, right panels).

We next examined combination treatment in a more clinically relevant setting, using CFZ rather than BTZ as it displayed a superior cytotoxicity profile in combination experiments (Figure 1). Twelve primary bone marrow aspirates from patient‐derived mononuclear cells (MNCs) were isolated from AML patients and grown in the HS‐5 co‐culture system. Patient‐derived cells were treated with DMSO or MKC8866. Similar to AML cell lines, treatment with MKC8866 did not affect primary AML cell viability (Figure 2A). However, there was increased cell death in AML patient‐derived MNCs when they were treated with CFZ in combination with MKC8866 (Figure 2B). As the CD34^+^ CD38^−^ cells are responsible for resistance and relapse, the impact of combination treatments on this population was examined. MKC8866 enhanced CFZ‐mediated cytotoxicity in the CD34^+^ CD38^−^ population (Figure 2C) as well as increasing the anti‐clonogenic effects of CFZ (Figure 2D). Active IRE1α‐XBP1s signalling can contribute to regulation of cytokines and chemokines, many of which can influence AML cell behaviour.^6^, ^7^ We investigated the impact of combination treatment on the expression of 12 disease‐relevant cytokines and chemokines using Evidence Evolution® Cytokine Array multiplexed assay. Levels of interleukin‐6 (IL‐6) were decreased in MKC8866‐treated samples while levels of monocyte chemoattractant protein (MCP‐1) were reduced in the AML patient co‐cultures when they received MKC8866 and were further decreased in samples receiving combination treatments (Figure 2E,F).

**FIGURE 2:**
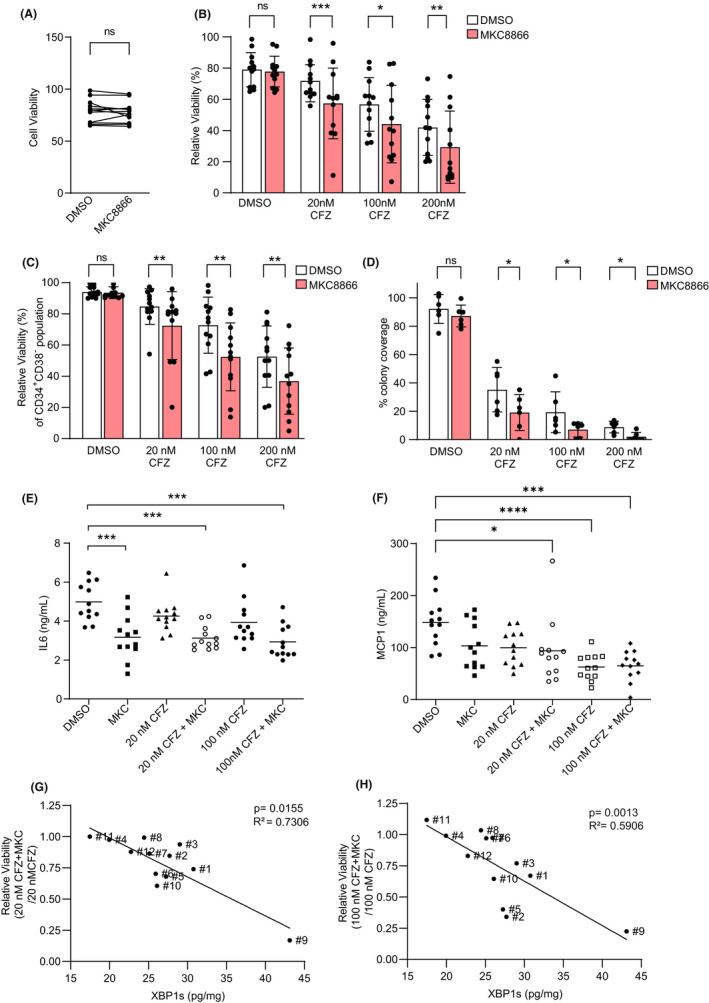
IRE1α RNase inhibition enhances CFZ cytotoxicity in CD34^+^ CD38^−^ population and XBP1s levels are predictive of combination response. 12 primary AML MCN samples (#1‐#12) were seeded on a layer of HS‐5 feeder cells that were tagged with the cell tracker dye carboxyfluorescein succinimidyl ester (CFSE). Before treatment, cells were grown in co‐culture for 24 h, after which the cultures were exposed to treatments. (A) Primary AML MNCs were treated with DMSO or MKC8866 for 72 h and cell viability was measured. (B) Primary AML bone marrow aspirates were co‐cultured with CFSE‐tagged HS‐5 cells for 24 h followed by 72 h treatment with the indicated agents. Total viable population of each sample was assessed after treatment before normalization to untreated sample. (C) Primary AML bone marrow aspirates were co‐cultured with CFSE tagged HS‐5 cells for 24 h followed by 72 h treatment with the indicated agents. CD34^+^CD38^−^ sub‐populations of these samples were assessed for viability using appropriate gating and flow cytometry followed by normalization to mean of untreated samples. (D) AML cells lines were cultured in HS‐5 conditioned media with supplements and methylcellulose for 14–21 days. A live cell confluency mask was used to quantify colony area coverage relative to untreated samples. Colony formation was quantified in AML patient samples #5, #8, #9, #10, #11 and #12. (E, F) Media from co‐cultures treated with indicated compounds were assessed for levels of IL‐6 and MCP‐1 using a high sensitivity cytokine biochip array. (G) Relative viability ratio (Relative viability upon 20 nM CFZ + MKC8866 co‐treatment ÷ Relative viability upon 20 nM CFZ treatment) was plotted against XBP1s levels. (H) Relative viability ratio (Relative viability upon 100 nM CFZ + MKC8866 co‐treatment ÷ Relative viability upon 100 nM CFZ treatment) was plotted against XBP1s levels. Bars = standard deviation. Statistical analysis was performed using Wilcoxon test, one‐way anova with post‐hoc Tukey test or Spearman's coefficient. ns = not significant, *p < 0.05, **p < 0.01, ***p < 0.001, ****p < 0.0001

XBP1s levels in MM patients positively correlate with patient outcome when treated with BTZ.^8^ No such correlative studies have been performed in AML. We compared basal XBP1s levels with viability of patient cells after combination treatment. XBP1s levels significantly correlated with MKC8866‐mediated enhancement of 20 nM and 100 nM CFZ treatment (Figure 2G,H). The cells that were most responsive to MKC8866 plus CFZ co‐treatment when compared with CFZ treatment alone had the highest XBP1s and vice versa.

This study proposes IRE1α‐XBP1s pathway inhibition as an adjunctive strategy for improving efficacy of proteasome inhibitors in AML. Proteasome inhibitors significantly increased XBP1s levels, and targeting the IRE1α‐XBP1s axis with MKC8866 in the presence of CFZ significantly enhanced cell death in AML cell lines and patient samples. We demonstrated this in a clinically relevant co‐culture model of AML, where resistance to therapy‐induced cell death is often an issue.

Although the AML cells used in this study constitutively expressed XBP1s, it was only in conditions of proteasome inhibitor‐induced upregulation of XBP1s that targeting this pathway provided therapeutic benefit. This suggests that while XBP1s may be basally active in AML cells, these cells are not reliant on XBP1s for survival. Despite this, basal levels of XBP1s in AML cells can prognosticate response to combination therapy and we demonstrate the utility of an XBP1 biochip to predict patient response. Sun et al. previously reported that IRE1α inhibition enhances the cytotoxic effects of BTZ in AML cell lines.^3^ In contrast to our findings, the same group demonstrated that targeting IRE1α alone decreased AML cell viability.^3^ Perhaps this was due to higher basal XBP1s in their cells and, therefore, enhanced sensitivity to IRE1 RNase inhibition, as a wide range of XBP1s mRNA levels has previously been reported in bigger AML cohorts.^9^ Notably, different IRE1α RNase inhibitors were also used, and off‐target toxicity could account for the differences observed.^3^

Acute myeloid leukaemia–stroma interactions have been implicated in promoting drug resistance and increasing the fraction of quiescent AML cells.^10^, ^11^ We demonstrate that CFZ and MKC8866 combination treatment can overcome HS‐5 protective effects. This co‐treatment also reduces proportional survival in the leukaemic stem cell (LSC)‐containing CD34^+^CD38^−^ population, an effect that has previously been shown to be stronger with CFZ than BTZ treatment.^11^ Signalling via the IRE1α‐XBP1s arm of the UPR preserves the self‐renewal capacity of pre‐LSCs and enables their clonal dominance over haematopoietic stem cells indicating an important role for XBP1s in LSC renewal.^12^ In this study, it is conceivable that LSC viability is decreased by the same mechanism and this contributes to the reduced clonogenic capacity observed with combination treatment. We also observe significant reductions in IL‐6 and MCP‐1 in combination treated cells, both of which impact haematopoietic stem cell phenotype.^13^, ^14^ These changes likely contribute to a more hostile environment for AML cells and contribute to increased AML cell death. Together, the results would suggest that combination treatment could improve patient outcomes and relapse rates.

In conclusion, we demonstrate the efficacy of a novel combination treatment using CFZ with MKC8866 in *ex vivo* AML models, to decrease the viability of AML cells. We propose that the combination treatment of CFZ and MKC8866 warrants further investigation as a treatment strategy for AML patients.

## AUTHOR CONTRIBUTIONS

**Stuart Creedican:** Data curation (lead); formal analysis (lead); investigation (lead); methodology (lead); writing – original draft (equal); writing – review and editing (equal). **Claire M. Robinson:** Formal analysis (equal); visualization (equal); writing – original draft (equal); writing – review and editing (lead). **Katarzyna Mnich:** Data curation (equal); investigation (equal); methodology (equal); writing – review and editing (equal). **Md Nahidul Islam:** Data curation (equal); investigation (equal); validation (equal); writing – review and editing (equal). **Eva Szegezdi:** Resources (equal). **Ruth Clifford:** Resources (equal). **Janusz Krawczyk:** Resources (equal). **John Patterson:** Resources (equal). **Stephen P. Fitzgerald:** Resources (equal). **Mark Summers:** Resources (equal). **Ciaran Richardson:** Resources (equal). **Kenneth Martin:** Conceptualization (equal); project administration (equal); resources (equal); supervision (equal); writing – review and editing (equal). **Adrienne M. Gorman:** Conceptualization (equal); funding acquisition (equal); project administration (equal); resources (equal); supervision (equal); writing – review and editing (equal). **Afshin Samali:** Conceptualization (equal); funding acquisition (equal); project administration (equal); resources (equal); supervision (equal); writing – review and editing (equal).

## CONFLICT OF INTEREST

AS, AG are co‐founders of Cell Stress Discoveries Ltd. KM, CR, MS are employed by Randox Teoranta. SPF is employed by Randox Holdings. JPP is co‐founder and employee of Orinove Inc.

## Data Availability

Data available on request from the authors
